# *In vivo* imaging of D_2_ receptors and corticosteroids predict behavioural responses to captivity stress in a wild bird

**DOI:** 10.1038/s41598-019-46845-x

**Published:** 2019-07-18

**Authors:** Christine R. Lattin, Devin P. Merullo, Lauren V. Riters, Richard E. Carson

**Affiliations:** 1 Department of Radiology and Biomedical Imaging, Yale University, New Haven, CT USA; 2 Department of Biological Sciences, Louisiana State University, Baton Rouge, LA USA; 3 Department of Integrative Biology, University of Wisconsin Madison, Madison, WI USA

**Keywords:** Positron-emission tomography, Stress and resilience

## Abstract

Individual physiological variation may underlie individual differences in behaviour in response to stressors. This study tested the hypothesis that individual variation in dopamine and corticosteroid physiology in wild house sparrows (*Passer domesticus*, n = 15) would significantly predict behaviour and weight loss in response to a long-term stressor, captivity. We found that individuals that coped better with captivity (fewer anxiety-related behaviours, more time spent feeding, higher body mass) had lower baseline and higher stress-induced corticosteroid titres at capture. Birds with higher striatal D_2_ receptor binding (examined using positron emission tomography (PET) with ^11^C-raclopride 24 h post-capture) spent more time feeding in captivity, but weighed less, than birds with lower D_2_ receptor binding. In the subset of individuals imaged a second time, D_2_ receptor binding decreased in captivity in moulting birds, and larger D_2_ decreases were associated with increased anxiety behaviours 2 and 4 weeks post-capture. This suggests changes in dopaminergic systems could be one physiological mechanism underlying negative behavioural effects of chronic stress. Non-invasive technologies like PET have the potential to transform our understanding of links between individual variation in physiology and behaviour and elucidate which neuroendocrine phenotypes predict stress resilience, a question with important implications for both humans and wildlife.

## Introduction

Within a species, individuals often display wide variation in behavioural responses to repeated and prolonged stressors^1,2^. For example, in response to cold weather^3^ and human disturbance^4^, some individual birds abandon their nests, while others continue breeding. These responses are often repeatable and appear to be somewhat heritable^5,6^, suggesting a possible physiological basis to these behaviours. Recent studies suggest that the catecholamine neurotransmitter dopamine may play a key role in stress-induced neuromodulation and some of the behavioural changes that result^7,8^. Although dopamine is best known for its role in reward-motivated behaviour^9^, two main pathways of the dopaminergic system also respond to stressful stimuli: the mesolimbic pathway connecting the ventral tegmental area to the nucleus accumbens, and the nigrostriatal dopamine pathway connecting the substantia nigra to the dorsal striatum^10,11^. Together with dopamine’s role in responding to rewards, this responsiveness to stressors has led to a broader hypothesis about dopamine function: that valence and salience coding by heterogeneous populations of dopamine neurons may be essential for learning which stimuli predict positive and negative outcomes and producing an appropriate behavioural response^12^.

Although a rise in dopamine may be important for learning about and responding appropriately to threats, laboratory animal and human studies suggest that neurobiological mechanisms limiting the extent and duration of dopamine effects on the brain may contribute to stress resilience. For example, individual rats resilient to the anhedonic effects of chronic stress displayed increased dopamine D_2_-type receptor density in mesolimbic dopamine circuits compared to stress-reactive individuals^13^, whereas knocking out D_2_ receptors in mice caused increased anxiety-like and depressive behaviours in response to stress^14^. In mammals, D_2_-type receptors (hereafter, “D_2_ receptors”) primarily act via two different mechanisms^15^. D_2_ receptors can be expressed postsynaptically on dopamine target cells, which upon binding inhibit second messenger activity and modulate target cell response. D_2_ receptors can also be found presynaptically on dopamine neurons, where they are involved in autoreceptor functions, including inhibition of dopamine release. Despite intriguing evidence that higher concentrations of D_2_ receptors may contribute to stress resilience, there is surprisingly little work examining relationships between individual variation in dopamine physiology and behavioural responses to chronic stressors.

There is also evidence that corticosteroid hormones (cortisol or corticosterone, depending on the species) are involved in mediating behavioural responses to stress. At baseline concentrations, corticosteroids help regulate metabolism, cognition, and immune function^16^. Corticosteroid concentrations increase dramatically in response to environmental challenges, and cause a number of physiological and behavioural changes designed to suppress non-essential functions and re-establish homeostasis^17^. These changes can include increased mobilization of energy stores, increased feeding behaviour, and suppression of reproductive behaviour^18^. Individual songbirds secreting more corticosteroids in response to a standardized (cloth bag) stressor also demonstrated increased behavioural responses to a predator model^19^, and were more likely to abandon their nests in response to low food availability and harsh weather^3^. Administering exogenous corticosteroids to free-living birds has also been shown to delay the return to breeding after a storm and to cause nest abandonment^20,21^.

This study tested the hypothesis that individual variation in dopamine and corticosteroid physiology in a wild songbird, the house sparrow (*Passer domesticus*), would significantly predict behaviour and weight loss in response to a long-term stressor, captivity. There is increasing evidence that despite unlimited access to food, wild animals continually perceive a captive environment to be stressful, and may never fully habituate to captivity^22^, although this may depend partly on individual and species differences^23^, and enrichment of the captive setting^24^. However, it is clear that the transition from the wild to captivity is highly stressful, as wild animals are exposed to stressors such as unpredictable contact with human caretakers^25^ and artificial lighting^26^. Recent work has also shown significant increases in DNA damage in wild birds brought into captivity^27^. In this study, all subjects were singly housed in large cages in a common room, and were all presumably stressed to some extent by captivity, but here we examined whether natural individual variation in physiology would be associated with better coping ability in the face of this chronic stressor.

**Figure 1:**
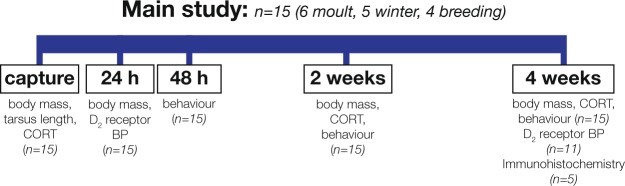
House sparrows (n = 15) were captured at three different times of year and samples for baseline corticosterone (CORT) collected in <3 min. Body mass and tarsus length were also measured, and birds exposed to a restraint stressor for 30 min, after which an acute stress CORT sample was taken. The day after capture, all animals underwent positron emission tomography (PET) scans with ^11^C-raclopride to quantify dopamine D_2_ receptor binding potential (BP). Two 1 h videos were recorded on the morning and the evening of the following day to assess behaviour. Body mass, baseline and acute stress CORT, and behaviour data were collected again 2 weeks and 4 weeks later. At 4 weeks post-captivity, a subset of animals (n = 11) underwent PET scanning again, and some of those (n = 5) were used for immunohistochemistry.

In keeping with past studies, we predicted that 4 weeks of captivity would cause decreases in body mass and behavioural disruption (increased anxiety-related behaviours and decreased feeding behaviour)^28,29^, but that individual variation in physiology would alter the extent of these effects, and explain individual-level variation in the response to chronic stress. Specifically, we predicted that individuals with increased striatal dopamine D_2_ receptor binding and a reduced corticosteroid response to a standardized stressor (i.e., animals with physiological mechanisms potentially limiting neuroendocrine changes induced by stress) would maintain higher body mass, spend more time feeding, and less time engaged in anxiety-related behaviours compared to individuals with lower D_2_ receptor binding and a greater corticosteroid response to a standardized stressor. We also examined changes in body mass, behaviour, corticosteroids and D_2_ receptor binding potential (BP) over time (Fig. 1).

A longitudinal study design is necessary to investigate the natural physiological variation that exists in a population and observe how this predicts later behaviour. However, most standard techniques for studying the brain either involve euthanizing animals (e.g., immunohistochemistry) or involve invasive procedures that may themselves alter neural function (e.g., *in vivo* microdialysis). This study was made possible by using positron emission tomography (PET) imaging with the D_2_ antagonist ^11^C-raclopride to quantitatively and noninvasively assess D_2_ receptor BP *in vivo*. Although researchers have used ^18^F-fluorodeoxyglucose PET in wild animals to examine neuronal activity^30–32^, no previous studies have taken advantage of PET’s unique ability to quantitatively assess specific neurotransmitter systems in living wild animals, and then observe how physiology predicts later behaviour.

## Results

### Test-retest reproducibility and displacement using haloperidol

**Figure 2:**
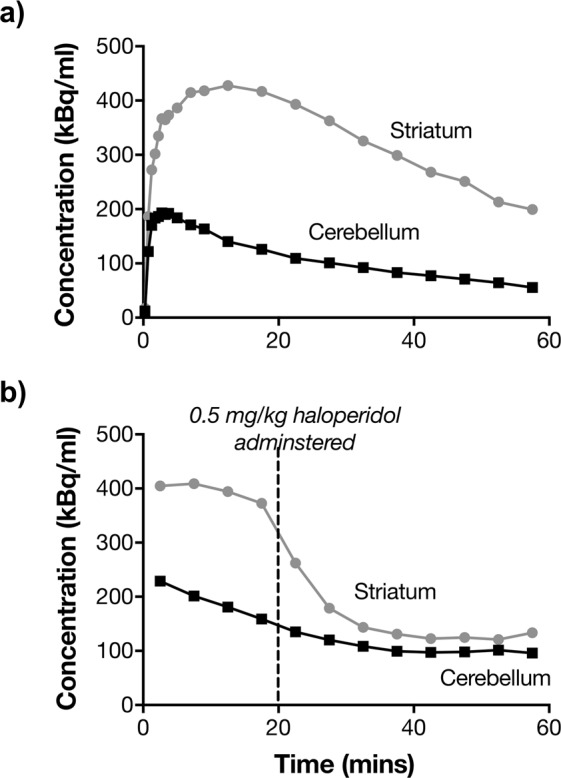
Baseline (**a**) and displacement (**b**) curves of ^11^C-raclopride in house sparrow striatum and cerebellum. During the displacement scan, a 0.5 mg/kg dose of haloperidol was administered 20 min after the start of the scan. For the displacement scan, the first 2.5 min of scan data were not captured.

Three individuals scanned twice one week apart showed good reproducibility in striatal D_2_ BP measures (relative test-retest variability = 0.4 ± 3.3%; absolute test-retest variability = 2.4%; intra-class correlation coefficient = 0.96). For comparison, recent ^11^C-raclopride human studies have reported intra-class correlation coefficients of 0.79–0.96 for caudate and putamen^33,34^. Baseline and haloperidol displacement curves for striatum and cerebellum indicated that a 0.5 mg/kg dose of the D_2_/D_3_/D_4_ antagonist haloperidol was able to displace nearly all of the specific binding of ^11^C-raclopride in striatum (Figs 2, S1).

### Changes in body mass, corticosterone, D2 receptor BP, and behaviour with time in captivity

Body mass varied by time period (capture vs. 24 h post-capture vs. 2 weeks post-capture vs. 4 weeks post-capture; Fig. S2, F_3,42_ = 32.76, p < 0.0001), and life history stage (moult vs. winter vs. breeding; F_2,42_ = 3.42, p = 0.042), but there was no significant effect of sex (F_1,43_ = 0.16, p = 0.69) or interaction between time period and life history stage (F_6,42_ = 1.78, p = 0.13). Post-hoc testing revealed that body mass at capture was higher than at any time in captivity (capture vs. 24 h post-capture: p < 0.0001; capture vs. week 2: p < 0.0001; capture vs. week 4: p < 0.0001) but body mass did not increase or decrease over 4 weeks of captivity (24 h post-capture vs. week 2: p = 0.96; 24 h post-capture vs. week 4: p = 0.47; week 2 vs. week 4: p = 0.75). Body mass was ~10% greater during winter compared to breeding (winter vs. breeding: p = 0.038; moult vs. breeding: p = 0.13; moult vs. winter: p = 0.96).

**Figure 3:**
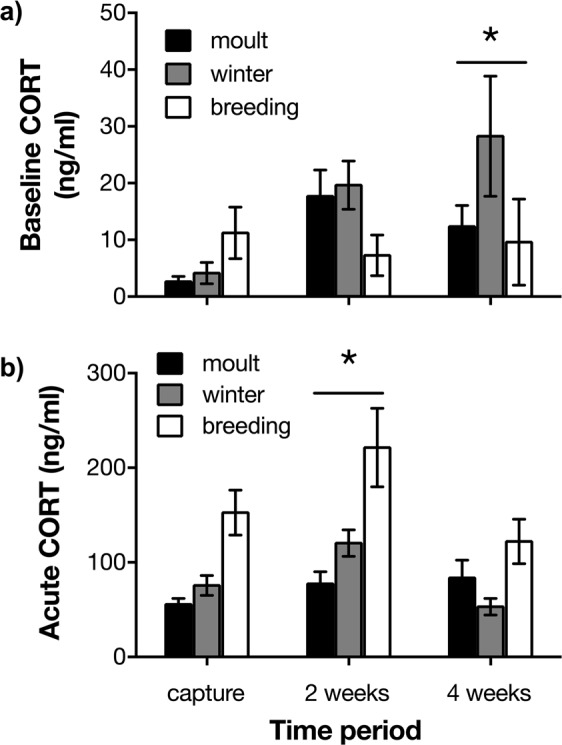
Plasma corticosterone (CORT) of wild house sparrows (*Passer domesticus*, moult: n = 6; winter: n = 5; breeding: n = 4) at capture and after 2 and 4 weeks in a laboratory setting: (**a**) at baseline (<3 min from time of capture or from entering the bird room), and (**b**) after 30 min of standardized restraint stress. Values are presented as mean ± SEM. Stars represent time periods with significantly increased hormone titres compared to capture (p < 0.05).

Baseline corticosterone varied by time period (capture vs. 2 weeks post-capture vs. 4 weeks post-capture; Figs 3, S3; F_2,23_ = 3.96, p = 0.033) but not by life history stage (F_2,33_ = 0.73, p = 0.49), or sex (F_1,11_ = 0.12, p = 0.74), and there was no interaction between time period and life history stage (F_4,23_ = 2.03, p = 0.12). Post-hoc testing revealed that baseline corticosterone was higher 4 weeks post-captivity compared to capture (capture vs week 2: p = 0.098; capture vs. week 4: p = 0.039; week 2 vs. week 4: p = 0.90). Corticosterone response to a cloth bag stressor also varied by time period (Figs 3, S3; F_2,24_ = 8.94, p = 0.0013), and females had higher acute corticosterone titres than males (F_1,10_ = 33.65, p = 0.0001). Acute corticosterone concentrations also varied by life history stage (F_2,30_ = 11.35, p = 0.0002), but there was no significant interaction between time period and life history stage (F_4,24_ = 2.74, p = 0.052). Post-hoc testing revealed that the acute stress response at week 2 was higher than at capture and week 4 (capture vs week 2: p = 0.0029; capture vs. week 4: p = 0.98; week 2 vs. week 4: p = 0.0033), and higher during breeding than moult or winter (breeding vs. moult: p = 0.0002; breeding vs. winter: p = 0.0052; winter vs. moult: p = 0.50).

**Figure 4:**
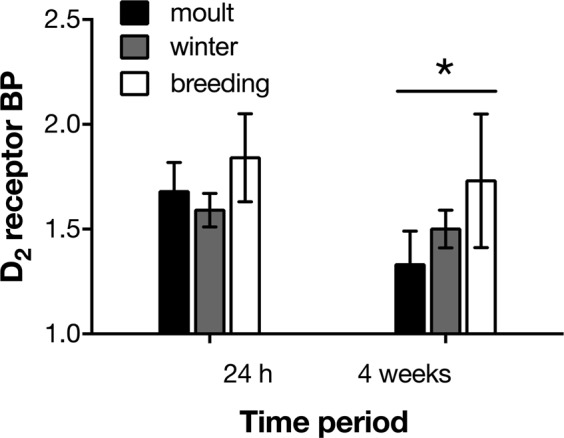
D_2_ receptor binding potential (BP) in striatum of house sparrows (*Passer domesticus*, moult: n = 6; winter: n = 5; breeding: n = 4) 24 h after capture and after 4 weeks in a laboratory setting. D_2_ receptor BP was assessed using 60 min positron emission tomography scans with ^11^C-raclopride; cerebellum was used as a reference region. Values are presented as mean ± SEM. The star indicates significantly decreased D_2_ receptor BP compared to 24 h post-capture (p < 0.05), although there was also a significant interaction between time period and life history stage (see Results for more details on statistical analyses).

In all birds imaged 24 h after capture (n = 15), there was no significant effect of initial life history stage on D_2_ receptor BP (F_2,14_ = 1.26, p = 0.32), although D_2_ receptor BP was higher in males than in females (F_1,14_ = 6.94, p = 0.023). In the subset of birds imaged twice (n = 11), D_2_ receptor BP varied by time period (24 h post-capture vs. 4 weeks post-capture), with 4-week captivity values lower than 24 h post capture values (Figs 4, S4; F_1,8_ = 9.39, p = 0.016). There was no significant effect of life history stage (F_2,7_ = 0.99, p = 0.42), or sex (F_1,7_ = 1.83, p = 0.22), but there was a time period by life history stage interaction (F_2,8_ = 10.14, p = 0.006), with birds captured during moult showing the greatest decrease in D_2_ receptor BP compared to other life history stages (moult 24 h post-capture vs. moult 4 weeks post-capture: p = 0.008; winter 24 h post-capture vs winter 4 weeks post-capture: p = 0.81; breeding 24 h post-capture vs. breeding 4 weeks post-capture: p = 0.70).

Neither baseline nor acute corticosterone titres at capture were significantly correlated with initial D_2_ receptor BP (r^2^ = 0.02, F_1,13_ = 0.32, p = 0.58 and r^2^ = 0.01, F_1,13_ = 0.14, p = 0.72, respectively). 4 weeks post-capture, we also did not see a relationship between D_2_ BP and baseline (r^2^ = 0.09, F_1,9_ = 0.84, p = 0.38) or acute corticosterone concentrations (r^2^ = 0.02, F_1,9_ = 0.16, p = 0.70). The change in D_2_ BP also showed no relationship with changes in baseline (r^2^ = 0.006, F_1,9_ = 0.057, p = 0.82) or acute corticosterone concentrations (r^2^ = 0.0003; F_1,9_ = 0.0031, p = 0.96).

Compared to 48 h post-capture, beak wiping behaviour was increased 2 weeks post-capture, and remained high 4 weeks post-capture (Fig. S5, Table S1). Overall activity (total number of hops and flights) was higher 2 weeks post-capture compared to 48 h post-capture, but decreased by 4 weeks post-capture. No other behaviours changed over the 4 weeks of captivity.

### Effects of initial physiology on behaviour and body mass in captivity

D_2_ receptor BP assessed 24 h after capture was positively associated with increased time spent feeding 2 and 4 weeks later (Table 1). The change in BP from 24 h to 4 weeks (measured in a subset of birds) also predicted the amount of feather ruffling (Table 2). Specifically, a decrease in D_2_ receptor BP with time in captivity was associated with increased feather ruffling behaviour 2 and 4 weeks post-captivity. Lower D_2_ receptor BP 24 h after capture was associated with higher body mass 2 and 4 weeks post-captivity (Table 3). Using a model selection approach, initial D_2_ receptor BP was a significant effect in best-fit models for time spent feeding and feather ruffling, and a near significant effect (p = 0.060) in the best-fit model for body mass (Table S2).

**Table 1:** The effects of initial D_2_ receptor binding potential (BP; measured ~24 h post-capture), and baseline and acute stress corticosterone (CORT; measured immediately after capture), on house sparrow (n = 15) behaviour ~48 h, and 2 and 4 weeks post-capture. Values represent *β* coefficients (in units of standard deviation), with standard errors in parentheses. The mixed model analysis included controls for initial life history stage, time of day, watcher, sex, and year, as well as nested 2-way random effects at the bird level and video level. Values in bold and with * indicate significance at p < 0.05.

	Behaviour				
	Beak wiping	Feather ruffling	Preening	Hops and flights	Feeding
Initial D_2_ receptor BP on 48 h behaviour	0.14 (0.33)	−0.32 (0.20)	0.21 (0.27)	−0.092 (0.27)	0.33 (0.19)
Initial D_2_ receptor BP on 2 week behaviour	0.056 (0.33)	−0.21 (0.20)	0.19 (0.28)	0.0066 (0.27)	0.64* (**0.20)**
Initial D_2_ receptor BP on 4 week behaviour	0.24 (0.33)	−0.055 (0.20)	0.076 (0.28)	−0.062 (0.27)	0.56* (**0.19)**
Initial baseline CORT on 48 h behaviour	0.36 (0.27)	0.68* (**0.17)**	0.16 (0.24)	0.062 (0.23)	−0.27 (0.18)
Initial baseline CORT on 2 week behaviour	0.40 (0.31)	0.77* (**0.23)**	0.57 (0.31)	−0.36 (0.28)	0.075 (0.24)
Initial baseline CORT on 4 week behaviour	0.30 (0.27)	0.49* (**0.17)**	0.20 (0.24)	0.18 (0.23)	0.14 (0.18)
Initial acute stress CORT on 48 h behaviour	−0.13 (0.34)	−0.098 (0.20)	−0.15 (0.29)	0.10 (0.27)	0.49* (**0.20)**
Initial acute stress CORT on 2 week behaviour	−0.077 (0.35)	−0.012 (0.22)	−0.22 (0.31)	−0.037 (0.29)	0.56* (**0.22)**
Initial acute stress CORT on 4 week behaviour	−0.045 (0.34)	−0.042 (0.21)	0.0090 (0.29)	0.046 (0.27)	0.28 (0.20)

**Table 2:** Model of effect of physiological changes (Week 4-Week 0, n = 11) on five different behaviours in captive wild house sparrows. The model included controls for life history stage, time of day, week of captivity, watcher, sex and year, and nested 2-way random effects at the bird level and video level. Values represent *β* coefficients (in units of standard deviation), with standard errors in parentheses. Corticosterone (CORT) was measured immediately after capture, and after 4 weeks in captivity. Dopamine D_2_ receptor binding potential (BP) was measured 24 h and 4 weeks after capture. Values in bold and with * indicate significance at p < 0.05.

	Behaviour				
	Beak wiping	Feather ruffling	Preening	Hops and flights	Feeding
D_2_ receptor BP change Week 4 to Week 0	−1.60 (2.61)	−4.47* (**1.29)**	−2.58 (1.52)	0.24 (0.96)	0.87 (1.63)
Baseline CORT change Week 4 to Week 0	0.75 (0.51)	0.69* (**0.26)**	0.62* (**0.30)**	−0.034 (0.19)	−0.25 (0.32)
Acute stress CORT change Week 4 to Week 0	0.94 (1.73)	2.67* (**0.85)**	1.90 (1.01)	−0.55 (0.64)	−0.74 (1.078)

**Table 3:** The effects of initial D_2_ receptor binding potential (BP; measured ~24 h post-capture), and baseline and acute stress corticosterone (measured immediately after capture), on house sparrow (n = 15) body mass ~24 h, and 2 and 4 weeks post-capture. The effects of initial body mass and tarsus length, two measures of initial bird body size, are also shown. Values represent *β* coefficients (in units of standard deviation), with standard errors in parentheses. The mixed model analysis included controls for initial life history stage, sex, and year, as well as random effects at the bird level. Values in bold and with * indicate significance at p < 0.05.

	Body mass
Initial D_2_ receptor BP on 24 h post-capture body mass	−0.37 (0.23)
Initial D_2_ receptor BP on 2 week post-capture body mass	−0.48* (**0.23)**
Initial D_2_ receptor BP on 4 week post-capture body mass	−0.55* (**0.23)**
Initial baseline CORT on 24 h post-capture body mass	−0.34 (0.22)
Initial baseline CORT on 2 week post-capture body mass	−0.28 (0.22)
Initial baseline CORT on 4 week post-capture body mass	−0.20 (0.22)
Initial acute stress CORT on 24 h post-capture body mass	0.22 (0.23)
Initial acute stress CORT on 2 week post-capture body mass	0.48* (**0.23)**
Initial acute stress CORT on 4 week post-capture body mass	0.30 (0.23)
Initial mass effect	0.061 (0.22)
Tarsus length effect	0.73* (**0.18)**

Baseline corticosterone measured immediately after capture was positively associated with increased feather ruffling behaviour at every time point (48 h, 2 and 4 weeks post-capture) (Table 1). The change in baseline corticosterone in captivity also predicted the amount of both feather ruffling and preening behaviour (Table 2), where larger increases in baseline corticosterone were associated with larger increases in feather ruffling and preening 2 and 4 weeks post-capture. Initial baseline corticosterone did not predict body mass in captivity at any time point (Table 3). Using a model selection approach, initial baseline corticosterone was a significant effect in best-fit models for beak wiping, preening, feather ruffling and body mass (Table S2).

Acute stress corticosterone measured at capture was positively related to the amount of time birds spent feeding 48 h and 2 weeks post-capture, but not 4 weeks post-capture (Table 1). Increasing the corticosterone response to acute stress post-captivity also predicted the amount of feather ruffling 2 and 4 weeks post-captivity (Table 2), where larger increases in acute stress corticosterone were associated with larger increases in feather ruffling behaviour. Initial corticosterone response to the cloth bag stressor was positively correlated with body mass after 2 weeks in captivity (Table 3). Using a model selection approach, the change in acute corticosterone was a significant effect in best-fit models for overall activity, beak wiping, and feather ruffling (Table S2).

### Immunohistochemistry

Representative double-immunolabeled brain sections from house sparrow striatum, ventral tegmental area, and substantia nigra are shown in Fig. S6. The same patterns were seen in all 5 animals examined. Specifically, in striatum, density of D_2_-like labelled cells was very high, and D_2_-like labeled cells were surrounded by a dense mesh of TH-like labelled fibres. No co-localization of TH and D_2_ labelling was found in striatum, suggesting postsynaptic D_2_ receptor regulation of non-dopaminergic cells. Co-localization between D_2_ and TH occurred in ventral tegmental area and substantia nigra (possibly indicative of presynaptic D_2_ receptors with autoreceptor functions^28^); some cells were also singly-labelled for D_2_ and TH.

### Dopamine agonist trials

In a separate group of birds not used in the rest of the study (n = 6), dopamine agonist treatment significantly affected feather ruffling behaviour (Fig. S7, F_2,120_ = 20.31, p < 0.0001), with birds performing this behaviour less after the high dose quinpirole treatment compared to the low dose treatment or a vehicle control (high dose vs low dose: p < 0.0001; low dose vs. control: p = 0.99; high dose vs. control: p < 0.0001). Feather ruffling was also affected by time period post-injection (F_5,120_ = 3.92, p = 0.0025), and there was a time * treatment interaction (F_10,120_ = 2.56, p = 0.0077). Beak wiping was affected both by time period post-injection (F_5,121_ = 4.58, p = 0.0007) and treatment (F_2,121_ = 4.95, p = 0.0086), and there was a time * treatment interaction (F_5,121_ = 2.26, p = 0.019). Post-hoc testing revealed that birds performed less of this behaviour after the high dose of quinpirole compared to the control treatment (high dose vs low dose: p = 0.063; high dose vs. control: p = 0.0070; low dose vs. control: p = 0.89). Number of preens was also affected by treatment (F_2,121_ = 3.62, p = 0.030). Post-hoc testing showed that birds that received the high dose of quinpirole did more preening than those that received saline (high dose vs low dose: p = 0.11; high dose vs. control: p = 0.028; low dose vs. control: p = 0.97). Preening was not significantly affected by time post-injection and there was no significant interaction (time: F_5,121_ = 2.27, p = 0.052; time * treatment: F_10,121_ = 1.32, p = 0.23). The other two behaviours were unaffected by dopamine agonist treatment: hops and flights (time: F_5,121_ = 0.93, p = 0.46; treatment: F_2,121_ = 0.13, p = 0.88; time * treatment: F_10,121_ = 1.15, p = 0.33) and feeding (time: F_5,121_ = 1.37, p = 0.24; treatment: F_2,121_ = 0.10, p = 0.90; time * treatment: F_10,121_ = 1.00, p = 0.45).

## Discussion

In this study, we examined several physiological measures in wild house sparrows at or shortly after capture to determine whether individual variation in physiology would predict the subsequent response to captivity (a chronic stressor). Generally, we found that individual variation in physiology did predict coping ability in captivity. Specifically, individuals with lower baseline and higher acute stress corticosteroid titres at capture performed fewer anxiety-related behaviours, spent more time feeding, and maintained higher body mass. Birds with higher striatal D_2_ receptor binding at capture spent more time feeding in captivity than birds with lower D_2_ receptor binding. In the subset of individuals imaged a second time, D_2_ receptor binding decreased in captivity (post-hoc analysis revealed this drop occurred specifically in moulting birds), and larger decreases were associated with increased anxiety behaviours 2 and 4 weeks post-capture. Ideally, we would have used wild animal controls to assess the effects of captivity on physiology, where a group of animals would have been caught, imaged, released, and then re-captured and re-imaged at the same time points as our captives. However, this was not feasible for this study – the risk of not being able to recapture animals we had previously imaged was too high.

Our initial hypothesis was that *lower* corticosteroids in response to a standardized acute stressor would be associated with stress resilience, but instead we found that individuals that coped better with the stress of captivity had lower baseline corticosterone, but *higher* acute stress corticosterone, at capture. This adds to a growing body of literature showing that baseline and acute stress corticosteroids can act in distinct ways^16,35^, and that the ability to mount a high acute corticosterone response to stress may reflect an individual’s stress resilience^36^. Our findings that higher capture levels of baseline corticosterone and increases in both baseline and acute stress corticosterone in captivity were associated with increased anxiety behaviours is in keeping with associations between elevated corticosteroids and increased anxiety-like behaviours observed in species from rodents^37^ to fish^38^ to birds^39^. The relationship between corticosteroids and feeding behaviour is more complicated, with some studies linking long-term increased corticosteroids to hyperphagia and others to hypophagia^29,40^. In songbirds specifically, exogenous corticosterone has been found to increase the number of food dish visits^41^ (although in another study, it only did so in fasting birds^42^); these findings are generally consistent with our results showing that elevated acute stress corticosterone at capture was associated with increased feeding behaviour in the lab.

We also predicted that higher D_2_ receptor BP would be associated with better coping. Interestingly, birds with higher D_2_ receptor BP 24 h after capture spent more time feeding in captivity, but actually weighed less, than birds with lower D_2_ receptor BP. The dopamine system plays a strong role in feeding; for example, transgenic mice that do not synthesize dopamine die of starvation because a lack of motivation to eat, which can be rescued by restoring striatal dopamine neurotransmission^43^. However, past rodent studies have also showed complicated and discrete effects of D_2_ receptor stimulation on feeding behaviour vs. food ingestion. For example, rats given the highly selective D_2_ agonist N-0437 showed no change in feeding duration, but did reduce food intake^44^. The D_2_ agonist PHNO actually increased food pellet consumption in rats, which was thought to be due to an increase in gnawing behaviour, because PHNO-treated rats decreased feeding on liquid diet, mash diet, and powdered diet^45^. Thus, D_2_ receptor stimulation seems to decrease food ingestion, but may actually increase some aspects of feeding behaviour. It is possible that sparrows with higher D_2_ receptor binding spent more time manipulating food (mostly seeds with shells, as well as food pellets), but actually consumed fewer calories, than sparrows with lower D_2_ receptor binding. We did not measure food ingestion in this study, so we cannot rule out this possibility. Our data are also consistent with laboratory rodent and human studies demonstrating higher D_2_/D_3_ receptor availability in lower-weight subjects^46,47^.

We also examined changes over time in physiological, behavioural, and body mass measures. As in other recent work, we saw decreased body mass, increased anxiety-related behaviours, and increased corticosteroid titres in captive sparrows^28^. Weight loss in particular has been shown to be a consistent indicator of chronic stress across many different stress paradigms and species, suggesting sparrows did indeed perceive the captive environment to be stressful^48^. *In vivo* PET imaging also allowed us to assess the effects of chronic captivity stress on D_2_ receptor BP. We found that compared to 24 h post-capture, individuals had reduced D_2_ receptor BP after 4 weeks of captivity, although this effect was only significant in post-hoc tests of moulting birds. Interestingly, we saw no initial difference in D_2_ receptor BP among moulting, winter, and breeding birds; this difference only emerged after 4 weeks of captivity. Examination of corticosterone data (e.g., in Fig. 3) suggests there was also seasonal variation in how hormone titres changed in captivity. Further exploring how seasonal variation in dopamine and corticosteroid systems interacts with chronic stressors would be a fruitful direction for future work, but unfortunately was beyond the scope of the present study due to low sample sizes across different life history stages. One potential limitation of D_2_ receptor BP is that it can be affected both by D_2_ receptor number and the amount of dopamine in the synapse; however, changes in dopamine level need to be large to affect the PET signal^49^. Regardless, our data suggest changes in dopaminergic systems could be one physiological mechanism underlying negative behavioural effects of chronic stress.

There is evidence that stereotyped, repetitive behaviours in captive animals and humans are at least partly due to disruptions in mesolimbic and nigrostriatal dopamine systems^7,50^. In fact, repeated injections of the D_2_/D_3_ receptor agonist quinpirole (which cause downregulation of D_2_ receptors^51^) are sometimes used to induce compulsive behaviours in rodents as a model for human obsessive-compulsive disorder^52^. We found larger decreases in D_2_ receptor binding in captivity associated with increased feather ruffling behaviour 2 and 4 weeks post-captivity. The functional role of D_2_-like receptors in anxiety behaviours was also demonstrated by our data showing that administration of a D_2_/D_3_ receptor agonist decreased both beak wiping and feather ruffling in a time- and dose-dependent manner, while leaving overall activity and time spent feeding unaffected. Overall, our results support the idea that dopaminergic disruption is a factor in the development of compulsive and stereotyped behaviours - which may be exacerbated or even triggered by chronic stress^53^.

Using immunohistochemistry with D_2_-like and TH-like double immunolabeling, we also provide some insight into potential mechanisms for how D_2_ receptors might regulate dopamine activity in sparrows. D_2_ receptors in sparrow striatum appear to be postsynaptic, although we did see evidence (i.e., co-labeled cells for D_2_ receptor and TH) for presynaptic autoreceptors in ventral tegmental area and substantia nigra, which project dopamine neurons into the avian striatum^54^. Presynaptic D_2_ autoreceptors in midbrain and postsynaptic D_2_ receptors in striatum is similar to what has been seen in mammals^55^, although this is the first time to our knowledge that D_2_ and TH proteins have been shown to co-localize in VTA and substantia nigra in songbirds. The pattern of double labelling is also consistent with a previous study demonstrating that neurons in songbird VTA and substantia nigra are autoinhibited by D_2_ dopamine receptors^56^. Overall, this work provides further evidence for conservation of mesolimbic and nigrostriatal dopamine pathways in birds and mammals.

Non-invasive technologies like PET are a valuable tool to determine whether individual variation in neuroendocrine phenotypes can successfully predict stress resilience, a research question with important implications both for human health and wild populations. However, high costs and technical challenges are still barriers to widespread use of PET imaging and mean that most published studies have low sample sizes^57^, including the present study. However, despite low sample size, we found that not only did *in vivo* PET imaging allow us to assess D_2_ receptor binding 24 h after capture and then show that our BP measure significantly predicted feeding behaviour and body mass in captivity, it also allowed us to demonstrate that this measure itself changed with time in captivity, and that the degree of this change predicted the amount of an anxiety-related behaviour birds performed. Understanding the physiological basis for individual variation in behavioural responses to chronic stressors will help us predict which individual animals will be the winners and losers in the face of anthropogenic stressors, and potentially help mitigate these effects.

## Methods

### Study subjects

Birds were brought into the lab during three different life history stages to capture the widest range of natural variation in baseline and acute stress-induced corticosteroids, which vary seasonally in house sparrows^58^ (Fig. 1). Sparrows were caught using Potter traps at bird feeders in New Haven, CT, USA during moult (13–21 Oct 2015 and 28–29 Sept 2016, n = 3 males and 3 females), winter (10 Nov–20 Dec 2016, n = 3 males and 2 females), and breeding (3 May–17 May 2017, n = 2 males and 2 females). All “moulting” birds were moulting flight feathers (P5 or higher). All “breeding” birds showed brood patches (females) or cloacal protuberances (males). Samples were taken >3 h after sunrise into the early afternoon (10 am–3 pm). Laboratory studies show that baseline corticosterone in captive house sparrows is uniform throughout this period^59^, and we found no relationship between time of capture and baseline corticosterone titres (r^2^ = 0.11, p = 0.25). At capture, 60 μl blood samples were collected from the alar vein. Blood samples collected <3 min after the door of the Potter trap closed were considered representative of baseline corticosterone^60^. Birds were then exposed to a standardized restraint stressor where they were put into clean, breathable cloth bags for 30 min, after which 30 μl blood samples were taken for determination of acute stress corticosterone (=90 μl total blood drawn). Birds were also weighed using Pesola spring scales and tarsus length measured with callipers.

In the lab, sparrows were singly housed with *ad libitum* access to mixed seeds, Purina LabDiet for small birds, grit, water, and sand for dustbathing. Day length in the lab corresponded to natural day length at capture, with light timing adjusted every two weeks (the day after measurements were taken). After 2 and 4 weeks in captivity, birds were re-weighed and additional blood samples collected for baseline and acute stress corticosterone. No-one entered the bird room 90 min prior to blood sampling. Animals were collected under Connecticut state permit 1417011, and all procedures carried out in accordance with protocol 2014–11648 approved by the Yale University Animal Care and Use Committee.

### Corticosterone assays

After centrifugation at 10,000 g, plasma was removed and stored at −80 °C. Plasma was assayed in duplicate wells using corticosterone ELISA kits (ADI-901-097, Enzo Life Sciences, Inc.)^61^. This assay has a sensitivity of 27 pg/ml. Samples were randomly allocated among plates, with baseline and acute stress corticosterone samples for each individual for each time point on the same plate. The interplate coefficient of variation was 9.8% and the intraplate coefficient of variation was 2.5% (calculated using replicates of pooled house sparrow plasma included on each plate).

### ^11^C-raclopride PET scans

^11^C-raclopride was prepared as previously described^62^. Radiochemical purity of the final product was >95%. Mean injected mass of raclopride was 0.016 ug (range: 0.0025–0.0844 ug). Neither injected mass of raclopride nor body mass-corrected injected mass was significantly correlated with D_2_ receptor BP (injected mass by D_2_ receptor BP: r^2^ = 0.008, p = 0.59, body mass-corrected injected mass by D_2_ receptor BP: r^2^ = 0.008, p = 0.60). All animals that were part of the main study (n = 15, Fig. 1) underwent PET scanning ~24 h after capture. Eleven of the 15 study subjects (moult: n = 3 males and 1 female; winter: n = 3 males and 1 female; and breeding: n = 2 males and 1 female) were scanned a second time, after 4 weeks of captivity (not all subjects could be re-scanned due to scanner issues). Animals were anesthetized using 4–5% isoflurane and imaged in an Inveon microPET-CT scanner (Siemens Medical Solutions USA). After induction, anaesthesia was lowered to 1–3% and temperature maintained with a heating pad. Injections were administered via an intraosseous catheter into the tibiotarsus at the beginning of the PET scan. We imaged the whole body for 60 min followed by a ~7 min CT scan for attenuation correction and drawing regions of interest.

### PET validations

Baseline-displacement experiments were conducted with the D_2_/D_3_/D_4_ receptor antagonist haloperidol to demonstrate the specificity of the D_2_ receptor binding signal. One male house sparrow not used in the main study underwent two PET scans one week apart: a baseline scan with ^11^C-raclopride, and a displacement scan one week later where 0.5 mg/kg haloperidol in sterile ethanolic saline was administered via intraosseous catheter 20 min after ^11^C-raclopride injection and the start of scanning. For the displacement scan, the first 2.5 minutes of scan data were not captured.

To assess test-retest reproducibility of BP measures, we re-imaged three of the animals from the main study >2 months after capture and compared BP values from these last two scans (scans 3 and 4, which were 1 week apart). Results were evaluated according to three different criteria: relative test-retest variability (TRV), absolute test-retest variability (aTRV), and intra-class correlation coefficient (ICC), as described previously^63^. The mean of TRV indicates the presence of a trend between the two scans, and the standard deviation of TRV is an index of the variability of the percent difference of two estimates. aTRV combines these two effects; in the absence of a between-scan trend, aTRV is comparable to the percent error in a single measurement. ICC is very similar to repeatability as defined by Lessells and Boag^64^, although the value of ICC can range from -1 (no reliability, BSMSS = 0) to 1 (identity between test and retest, WSMSS = 0)^65^.

### PET reconstructions and PET image analysis

**Figure 5:**
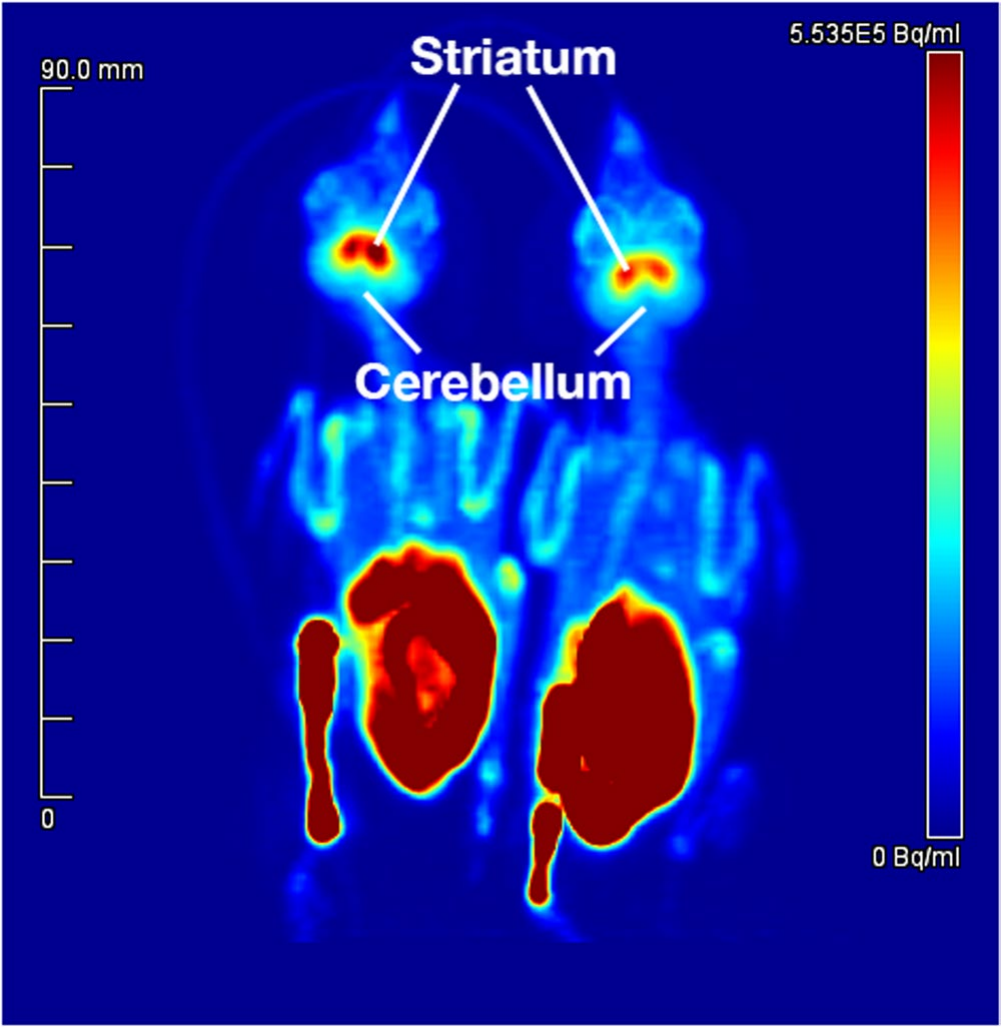
Maximum intensity projection image of a positron emission tomography (PET) scan in two house sparrows (*Passer domesticus*). ^11^C-raclopride was administered into the tibiotarsus of anesthetized animals and PET data collected for 60 min. PET data were reconstructed for the entire time frame using a vendor-supplied 3D OSEM/MAP algorithm with computed tomography attenuation and scatter correction applied to the data. The locations of the striatum and cerebellum are noted.

PET images were reconstructed as follows: 1) static images that summed data from the entire 60 min PET scan (Fig. 5), and 2) dynamic images that binned data into 16 time frames (8 × 30 s, 3 × 2 min, and 5 × 10 min). Reconstructions used a vendor-supplied 3D OSEM/MAP algorithm with CT attenuation and scatter correction applied to the data. We used 2 OSEM iterations, 16 subsets and 18 MAP iterations and a beta of 0.0023, identified as providing full width at half maximum (FWHM) resolution of ~1 mm^66^. Images were analysed using Inveon Acquisition Workplace software (Siemens Healthcare GmbH). As performed previously^31^, we used a digital canary brain atlas^67^ to define regions of interest corresponding to cerebellum and striatum. Briefly, CT images of canary and house sparrow skull were aligned using automated rigid registration tools, then canary atlas ROIs were brought into alignment with co-registered sparrow PET-CT images and applied to the sparrow PET image.

Time–activity curves were fit using the simplified reference tissue model (SRTM)^68^ and a graphical analysis (GA) approach^69^. BP (also denoted BP_ND_) was calculated for each model by averaging values from left and right brain structures. BP values determined from the two different models was highly correlated (r^2^ = 0.99, p < 0.0001), so SRTM values were used for subsequent analyses.

### Behavioural analysis

The day after PET scanning (i.e., ~48 h after capture), and then again 2 and 4 weeks after capture, one hour of video was recorded for each bird 1 h after lights on and a second hour of video recorded 2 h before lights off. The camera was turned on and off remotely, and no-one entered the room for 30 min preceding the start of recording. Videos were analysed for five different behaviours: time spent feeding, and number of hops and flights, preens, beak wipes, and feather ruffles. Beak wiping and feather ruffling have been associated with anxiety in house sparrows and other birds^28,39,70^. We measured all behaviours as previously described^28^. Behaviours were scored by three observers blinded to time period, and videos were watched in random order. Behavioural measures were highly repeatable within and between different observers (SI Methods). These five behaviours are not strongly correlated in captive house sparrows^28^, so we chose to analyse them separately rather than combining them using principle components analysis.

### Dopamine agonist trials and immunohistochemistry

To investigate behavioural effects of pharmacological manipulation of the dopamine system, in another set of animals not used in the main study (n = 3 males and 3 females, caught in December 2016 and kept in captivity ~8 weeks before beginning the experiment), we assessed the short-term effects of administering a D_2_/D_3_ receptor agonist, quinpirole. Quinpirole doses were based on a previous study showing significant effects on male Japanese quail sexual behaviour^2^. Quinpirole (Q102, Sigma Aldrich) was dissolved in saline, or saline only given as a control, and all injections administered subcutaneously over the pectoralis muscle. Animals received two different doses of quinpirole: 0.1 mg/kg (the “low dose”), and 1 mg/kg (the “high dose”), as well as saline controls. Each subject was tested 4 times on 4 different days within a 1-week period. On days 1 and 4, all birds received a vehicle control. On day 2, half the birds received the high dose of quinpirole and the others received the low dose, with the doses switched on day 3. Behaviour was video-taped starting immediately after injection, for 90 min (SI Methods). We also examined possible mechanisms for D_2_ receptor regulation of the dopamine system in songbird striatum by using double-fluorescent immunohistochemistry to visualize cells expressing TH-like and D_2_-like binding in striatum, ventral tegmental area, and substantia nigra^71^ (SI Methods).

### Statistical analyses

To assess changes in body mass, corticosterone, and D_2_ receptor BP with time in captivity, we applied Gaussian linear mixed-models using JMP Pro 13 (SAS Institute Inc., Cary, NC). These models included individual as a random effect to account for the repeated-measures nature of the data, and sex, time period (capture, 2 weeks post-capture, and 4 weeks post-capture), initial life history stage (moult, winter, or breeding) and time period * life history stage interaction fixed effects. The D_2_ BP model only included ~24 h post-capture and 4-week D_2_ receptor BP data, and only had data for 11 of the 15 individuals (see above). In the full dataset of 24 h post-captivity D_2_ receptor BP data (n = 15), we also assessed the effects of initial life history stage and sex on D_2_ receptor BP using general linear models, after testing for potential unequal variances among group residuals using Levene’s test. We used Tukey’s HSD post-hoc tests where appropriate.

We estimated the effect of time in captivity on behaviour as a linear mixed model using restricted maximum likelihood (“mixed” command in Stata 15.1, StataCorp LP, College Station, TX). This model incorporated video-level random effects, bird-level random effects, and an observer-level error term (because three different observers watched different subsets of videos). We regressed behaviour measures on variables indicating whether animals had been captive for 2 or 4 weeks. All analyses included controls for sex, year, life history stage, and time of day (videos were recorded both in the morning and evening). We assumed that bird- and video-specific effects were orthogonal to the other covariates of the model, and that residual errors were independent and identically normally distributed^72^. Standard tests for heteroscedasticity do not apply in the case of mixed models with random effects estimated using REML. Thus, to address concerns about whether heteroscedasticity might be causing incorrect standard errors, we re-estimated our behaviour model, replacing the bird random effects with fixed effects, and eliminating the video-level random effects. The model can then be estimated using OLS and the White test^73^. We did not reject the null hypothesis of homoscedasticity with p > 0.05 for any behaviour (data not shown). We tested the orthogonality of the random effects by conducting a Hausman test of the baseline random effects model compared with an alternate model of bird-level fixed effects^74^. For all outcomes we failed to reject the null hypothesis of the consistency of the random effects model (data not shown).

Our main study question was whether initial individual variation in physiology (i.e., baseline and acute stress corticosterone, and D_2_ receptor BP) would predict subsequent body mass and behaviour in captivity. To assess this, we used a model similar to the approach used to analyse the effect of time in captivity on behaviour, which added measures of initial physiology (baseline corticosterone, acute stress corticosterone, and D_2_ receptor BP) while retaining all other variables. Multiple physiological variables and time points were run in a single model in order to limit the risk of Type I error – i.e., for each of the 5 behaviours and body mass, we included 48 h, 2 week, and 4 week data, and baseline and acute corticosterone and D_2_ BP in the same model. Although sample sizes were relatively small, only two subjects per independent variable are required for adequate estimation of regression coefficients, standard errors, and confidence intervals in linear regression analyses^75^. Because regressions included random effects, standard errors were adjusted using Kenward-Roger corrections. The effects of initial physiological measures were allowed to vary depending upon time in captivity – e.g., we separately estimated the effects of initial D_2_ BP on behaviours 48 h, 2 weeks and 4 weeks after capture. Models with body mass as the dependent variable omitted observer fixed effects, time of day controls, and video-level random effects, and included initial body mass and tarsus length. The advantage of this kind of model is that it allows both for hypothesis testing and gives *β* coefficients, measured in units of standard deviation. *β* coefficient values are useful when trying to understand which independent variables have a greater effect on the dependent variable in a multiple regression analysis when the variables are measured in different units (in this case, ng/ml for corticosteroids, and unitless BP measures for D_2_ receptor binding). This also allowed us to assess whether the relationship between initial physiology and behaviour became stronger or weaker over time.

We also examined how the change in baseline and acute stress corticosterone and D_2_ BP between weeks 0 and 4 was associated with different behaviours in weeks 2 and 4. That is, did *the change in physiology* predict an animal’s behaviour in captivity? In these analyses, we used the model described just above, but redefined the independent variable as the difference in physiology from week 0 to week 4, and estimated the effect of this difference on the level of behaviour in weeks 2 and 4. All controls and random effects were the same as previously.

Many researchers in the field of physiological ecology have begun to move away from traditional null hypothesis testing and adopted a model selection approach^76^. Thus, as an alternative way to analyse our data, we used JMP Pro 14.1.0 to do model selection with penalized regression using the relaxed lasso method^77,78^ (SI Methods). For each outcome, the best-fit model was determined using AICc score. Finally, using JMP, we regressed baseline and acute stress corticosterone against D_2_ BP using initial values, 4 week post-captivity values, and the change from initial to post-captivity values, to examine data for potential correlations among different physiological variables. Values are presented as means ± SEM.

## Supplementary information

Lattin et al. Supplementary material

Lattin et al. raw data

## Data Availability

All data generated or analysed during this study are included in this published article and its Supplementary Information files.
