# Supplementary material for: In vivo imaging of D2 receptors and corticosteroids predict behavioural responses to captivity stress in a wild bird
Source: Sci Rep. 2019 Jul 18;9:10407. doi: 10.1038/s41598-019-46845-x (PMC6639298; doi:10.1038/s41598-019-46845-x)
Supplement: Supplementary file 1 — Lattin et al. Supplementary material [file 41598_2019_46845_MOESM1_ESM.docx]

**Supplementary Information for: I*n vivo* imaging of D_2_ receptors and corticosteroids predict behavioural responses to captivity stress in a wild bird**

**Christine R. Lattin, Devin P. Merullo, Lauren V. Riters, Richard E. Carson**

**SI Methods**

*Inter- and intra-observer repeatability of behavioural analysis*

Repeatability was calculated using variance components from an ANOVA^1^. Intra-observer repeatability (assessed by observers re-watching a subset of videos) was high: beak wiping=0.92; feather ruffling=0.96; feeding=0.99; hops and flights=0.99; preening=0.76. Inter-observer repeatability (assessed by two observers both watching the same subset of videos) was also high: beak wiping=0.98; feather ruffling=0.97; feeding=0.88; hops/flights=0.95; preening=0.99.

*Dopamine agonist trials*

One observer watched all videos and was blinded to treatment. Behaviours were analysed as described in the Methods (see “*Behavioural analysis*”), except that behaviours were binned into 15 min increments to account for potential differences in behaviour at different times post-injection. For statistical analysis, we applied Gaussian linear mixed-models using JMP Pro 14 with individual as a random effect and treatment (high quinpirole dose, low quinpirole dose, and vehicle control), time period (here, 15 min periods from 0 to 90 min post-injection, 0-15 min, 15-30 min, etc.), and their interaction as fixed effects. When we examined the residuals for each time*treatment interaction using Levene’s test, the variance in residuals across groups were not significantly different for hops and flights and time spent feeding (p>0.05). However, preening, feather ruffling, and beak wiping did not pass Levene’s test, so we log(x+1) transformed these data, which served to equalize the variance in the residuals across groups. We then ran analyses on the transformed data. One individual in the low quinpirole dose group was an outlier in feather ruffling behaviour during the first 15 min post-injection, so was excluded from this analysis (22 ruffles, compared to a mean of 2.3 ± 0.3 ruffles for all other individuals). Because there was no statistical difference in any behaviour between the first and second vehicle control trials (data not shown), we combined these data into a single "control" treatment group. We used Tukey's HSD post-hoc tests when appropriate.

*Immunohistochemistry*

Outside of mammalian species, much less is known about the possible presynaptic vs postsynaptic role of D_2_ receptors in modulating dopamine activity in striatum. To gain insight into potential mechanisms of D_2_ receptor regulation of the dopamine system in songbird striatum, we also performed double fluorescent immunolabeling to observe the degree to which co-localization patterns of tyrosine hydroxylase (TH, the rate-limiting enzyme in dopamine synthesis) and D_2_ receptors differed across three different brain regions in the nigrostriatal and mesolimbic pathways: the ventral tegmental area and substantia nigra of the midbrain, and the striatum. (Note that although in birds an area of the striatum specifically homologous to the mammalian nucleus accumbens is believed to exist, there is no precise criterion currently used to unambiguously delineate this region from the rest of the striatum^3^, so the avian striatum contains areas homologous to both the mammalian dorsal striatum and the nucleus accumbens.) We were specifically looking for anatomical evidence that sparrows have both presynaptic and postsynaptic D_2_-like receptors similar to what has been seen in mammals: co-localization of TH-immunoreactive and D_2_-immunoreactive cells in ventral tegmental area and substantia nigra (indicative of possible presynaptic autoreceptors) and segregation of TH-immunoreactive cells and D_2_-immunoreactive cells in striatum (indicative of postsynaptic D_2_-like receptors regulating the activity of nondopaminergic cells)^4^.

Six dopamine receptors have been cloned in zebra finch: D_1A_, D_1B_, D_1D_ (all within the D_1_-like family), and D_2_, D_3_, and D_4_ (all within the D_2_-like family)^5^. Their distribution shown using *in situ* hybridizations has revealed that the mesolimbic and nigrostriatal pathways (ventral tegmental area, substantia nigra, and striatum) are enriched in D_1A_, D_1B_, and D_2_ receptors. Five of the sparrows used in the main study (Fig 1; n=2 winter, 3 breeding) were anesthetized with ketamine (80 mg/kg b.w.) and xylazine (20 mg/kg b.w.) and transcardially perfused with ice-cold saline followed by 4% paraformaldehyde after their final PET scan. Tissue was sectioned using a cryostat into 40 µm thick coronal sections collected into antifreeze cryoprotectant solution. Regions were identified by anatomical features based on the zebra finch brain atlas^6^. Specifically, we used brain sections containing Area X for striatum and sections containing the third cranial nerve for substantia nigra and ventral tegmental area. The primary antibody used for D_2_ receptors was a D_2_ receptor antibody raised in rabbit (Millipore ab5084P; diluted 1:100). After incubation in HRP-conjugated goat anti-rabbit antiserum (catalogue No. 7074s, Cell Signaling Technologies, Danvers, MA; diluted 1:100), D_2_-like receptors were labelled red using Cy3-conjugated tyramide (catalogue No. NEL744001KT, TSA^TM^ Plus Cyanine 3 kit, PerkinElmer, Waltham, MA). The primary antibody for TH was raised in mouse (Millipore ab318; diluted 1:500). Following incubation in HRP-conjugated horse anti-rabbit antiserum (catalogue No. 7076s, Cell Signaling Technologies; diluted 1:100), TH was labelled green using Alexa Fluor 488-conjugated tyramide (catalogue No. B40932, Molecular Probes, Eugene, OR). Antibodies were validated for specificity in house sparrows using Western immunoblots to ensure that single dark bands were present at the appropriate molecular weights for each antibody (Fig S8). For D_2_, a single band appears at <100 kDa. Because there are not multiple bands, it does not appear that other subtypes (e.g., D_3_ and D_4_) are present. Although the manufacturer’s website states that the expected size is 50 kDa, they also note that several publications have seen bands at approximately 68 and 100 kDa (e.g.^7^). This band may represent the 92 kDa ligand-binding subunit of the D_2_ receptor.

Images were captured using a Zeiss LSM 780 Meta laser scanning confocal microscope and associated camera. Although we did not quantify cell counts, we examined images of striatum, substantia nigra and ventral tegmental area (two images of each area, one from the left and right hemispheres) across all 5 individuals and analysed labelling patterns.

*Model selection approach*

As an alternative to null hypothesis testing, we also analysed our data using model selection with penalized regression using the relaxed lasso method. Lasso methods can be used where there is the potential for overfitting a model; unlike stepwise model selection, lasso uses a tuning parameter to penalize the number of parameters in the model. Outcomes of interest were the five behaviours described above and body mass, and included 48 h post-capture, 2 week and 4 week data. For the behaviour models, we included all of the following possible model effects: video date, focal bird ID, sex, time post-captivity, initial life history stage, time of day the video was recorded, video ID, individual doing the observations, the bird’s initial body mass at capture, the bird’s present body mass, tarsus length, wing chord, D_2_ BP 24 h after capture, the change in D_2_ binding potential, baseline corticosterone at capture, the change in baseline corticosterone, acute stress corticosterone at capture, and the change in acute corticosterone. For the change in body mass, we excluded effects only related to behaviours (time of day video was recorded, video ID, and individual doing the observations), and the bird’s present body mass was used as the outcome. Best-fit model were determined using AICc scores.

**Table S1.** Behaviour of house sparrows (n=15) 2 and 4 weeks post-capture compared to initial (48 h post-capture) behaviour. Values represent $\beta$ coefficients (measured in units of standard deviation), with standard errors in parentheses. Mixed model analyses included controls for initial life history stage, time of day, watcher, sex, and year, as well as nested 2-way random effects at the bird level and video level. Values in bold and with * indicate significance at p<0.05.

|  | Behaviour | | | | |
| --- | --- | --- | --- | --- | --- |
| Time period | Beak wiping | Feather ruffling | Preening | Hops and flights | Feeding |
| 2 weeks post-capture | **1.1^*^ (0.17)** | -0.089 (0.19) | 0.15 (0.23) | **0.45^*^ (0.20)** | -0.081 (0.22) |
| 4 weeks post-capture | **1.3^*^ (0.17)** | -0.069 (0.18) | -0.066 (0.22) | 0.20 (0.20) | -0.41 (0.22) |

**Table S2.** Results from the single best fit penalized regression model (and associated AICc and r^2^ values) using the relaxed lasso approach for five different behaviors and body mass of house sparrows (n=15) in captivity. Values in table cells represent estimates (standard error), followed by Wald χ^2^ and p values. Bolded values with * represent significant effects. In cases where no p value is listed for a particular model effect, those effects were not present in the best-fit model. For body mass, cells with “---” were not used as potential model effects. CORT=corticosterone.

| Effect | Hops and flights  AICc: 212.1  r^2^=0.41 | Time feeding  AICc: 240.8  r^2^=0.39 | Beak wiping  AICc: 185.30  r^2^=0.59 | Preening  AICc: 267.2  r^2^=0.23 | Feather ruffling  AICc: 181.1  r^2^=0.65 | Body mass  AICc: 73.10  r^2^=0.52 |
| --- | --- | --- | --- | --- | --- | --- |
| Date |  |  |  |  | -4.63e-8 (3.24e-8)  χ^2^=2.04  P=0.15 | -3.65e-8 (1.04e-8)  **χ^2^=12.36***  **P=0.0004** |
| Focal bird ID | 0.037 (0.016)  **χ^2^=5.19***  **P=0.023** | 0.030 (0.031)  χ^2^=0.96  P=0.33 |  |  | -0.10 (0.056)  χ^2^=3.46  P=0.063 |  |
| Watcher |  |  |  |  |  | --- |
| Sex (F-M) | -0.32 (0.17)  χ^2^=3.68  P=0.055 |  |  | 0.48 (0.23)  **χ^2^=4.53***  **P=0.033** |  |  |
| Weeks post-captivity | 0-4: -0.40 (0.17)  2-4:0.20 (0.19)  **χ^2^=10.86***  **P=0.0044** |  | 0-4: -1.08 (0.13)  2-4: removed from model  **χ^2^=67.63***  **P<0.0001** |  |  |  |
| Initial life history stage | Breeding-winter: ‑0.34 (0.18)  Molt-winter: 0.95 (0.36)  **χ^2^=11.27***  **P=0.0036** |  | Breeding-winter: ‑0.47 (0.17)  Molt-winter: removed from model  **χ^2^=7.63***  **P=0.0058** |  | Breeding-winter: removed from model  Molt-winter: ‑1.67 (0.59)  **χ^2^=8.10***  **P=0.0044** | Breeding-winter: 1.41 (0.43)  Molt-winter: removed from model  **χ^2^=10.73***  **P=0.0011** |
| Time of day (evening-morning) | -0.71 (0.14)  **χ^2^=24.4***  **P<0.0001** | 0.83 (0.18)  **χ^2^=21.45***  **P<0.0001** |  | -0.47 (0.20)  **χ^2^=5.53***  **P=0.019** | -0.68 (0.12)  **χ^2^=32.07***  **P<0.0001** | --- |
| Initial body mass |  |  | 0.53 (0.11)  **χ^2^=25.48***  **P<0.0001** |  | -0.52 (0.20)  **χ^2^=6.65***  **P=0.0099** |  |
| Animal’s present body mass |  |  |  |  |  | --- |
| Tarsus length |  |  |  |  | -1.46 (0.44)  **χ^2^=10.96***  **P=0.0009** |  |
| Wing chord |  | -0.088 (0.041)  **χ^2^=4.60***  **P=0.032** | -0.083 (0.030)  **χ^2^=7.91***  **P=0.0049** |  | -0.07 (0.04)  χ^2^=2.55  P=0.11 |  |
| Video ID |  | -0.028 (0.014)  χ^2^=3.80  P=0.051 |  | -0.019 (0.0057)  **χ^2^=11.46***  **P=0.0007** |  | --- |
| Initial D_2_ binding potential |  | 0.28 (0.11)  **χ^2^=5.92***  **P=0.015** |  |  | -0.66 (0.14)  **χ^2^=21.70***  **P<0.0001** | -0.31 (0.092)  χ^2^=3.52  P=0.060 |
| Change in D_2_ binding potential (week 4-week 0) |  |  |  | 0.40 (0.11)  **χ^2^=12.40***  **P=0.0004** |  |  |
| Initial baseline CORT |  | -0.061 (0.088)  χ^2^=0.47  P=0.49 | 0.69 (0.11)  **χ^2^=41.92***  **P<0.0001** | 0.28 (0.10)  **χ^2^=7.38***  **P=0.0066** | 0.36 (0.12)  **χ^2^=8.68***  **P=0.0032** | -0.31 (0.092)  **χ^2^=11.51***  **P=0.0007** |
| Change in baseline CORT (week 4-week 0) |  |  |  |  |  |  |
| Initial acute CORT |  |  |  |  |  |  |
| Change in acute CORT (week 4-week 0) | -0.30 (0.048)  **χ^2^=6.87***  **P=0.0088** |  | 0.45 (0.079)  **χ^2^=31.65***  **P<0.0001** | 0.13 (0.22)  χ^2^=1.26  P=0.26 | 0.83 (0.20)  **χ^2^=17.84***  **P<0.0001** |  |


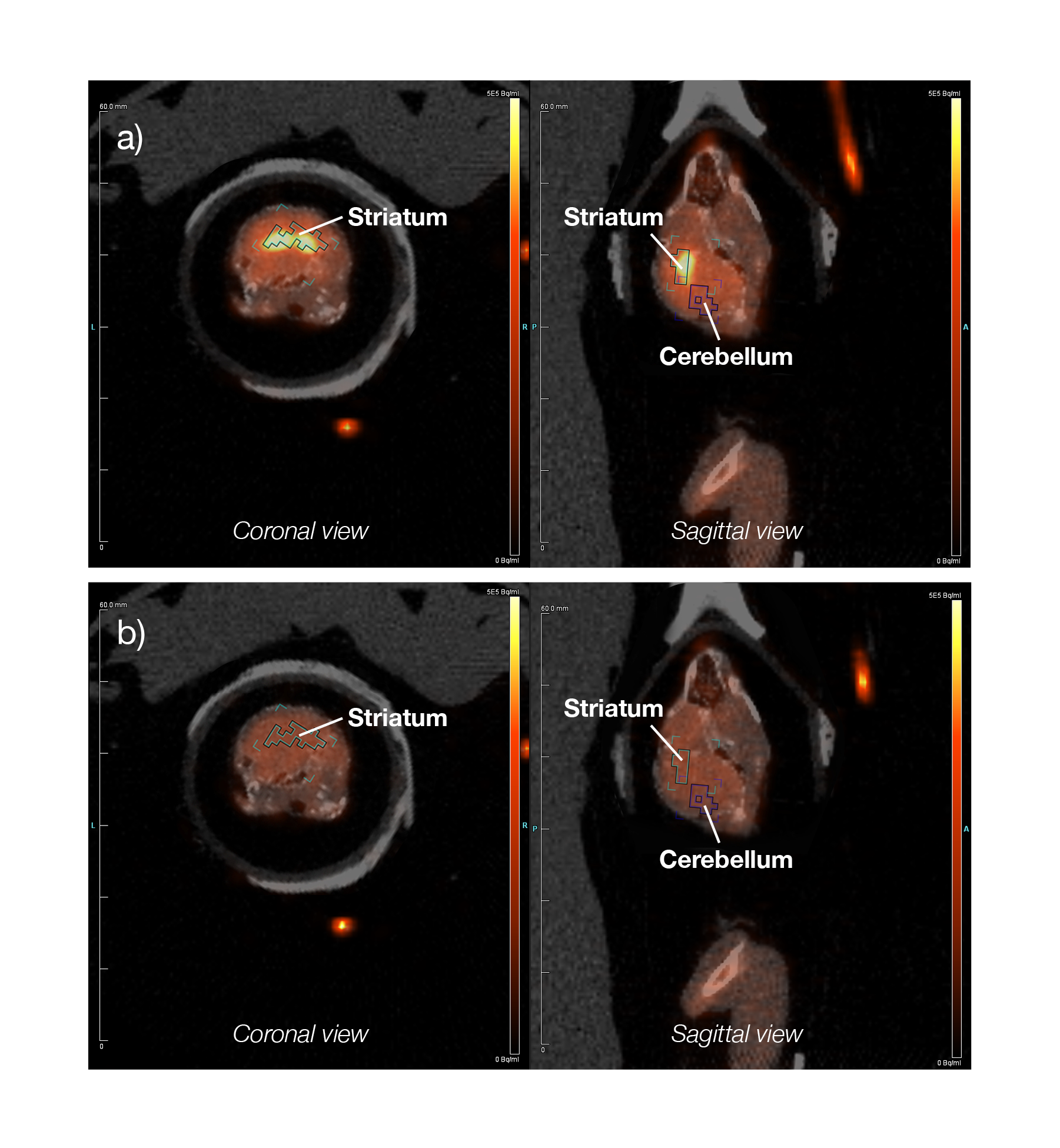


**Fig S1**. Positron emission tomography summed images (red scale) overlaid onto computed tomography images (grey scale) depicting ^11^C-raclopride (total injected mass: 0.0088 μg) in house sparrow striatum and cerebellum before a 0.5 mg/kg dose of haloperidol was administered (5-10 min, a) and after haloperidol administration (20-25 min, b). Haloperidol was administered 20 min after the start of the scan. The scale bar represents 60 mm, and the positron emission tomography red scale goes from 0 to 500 kBq/ml for both sets of images.


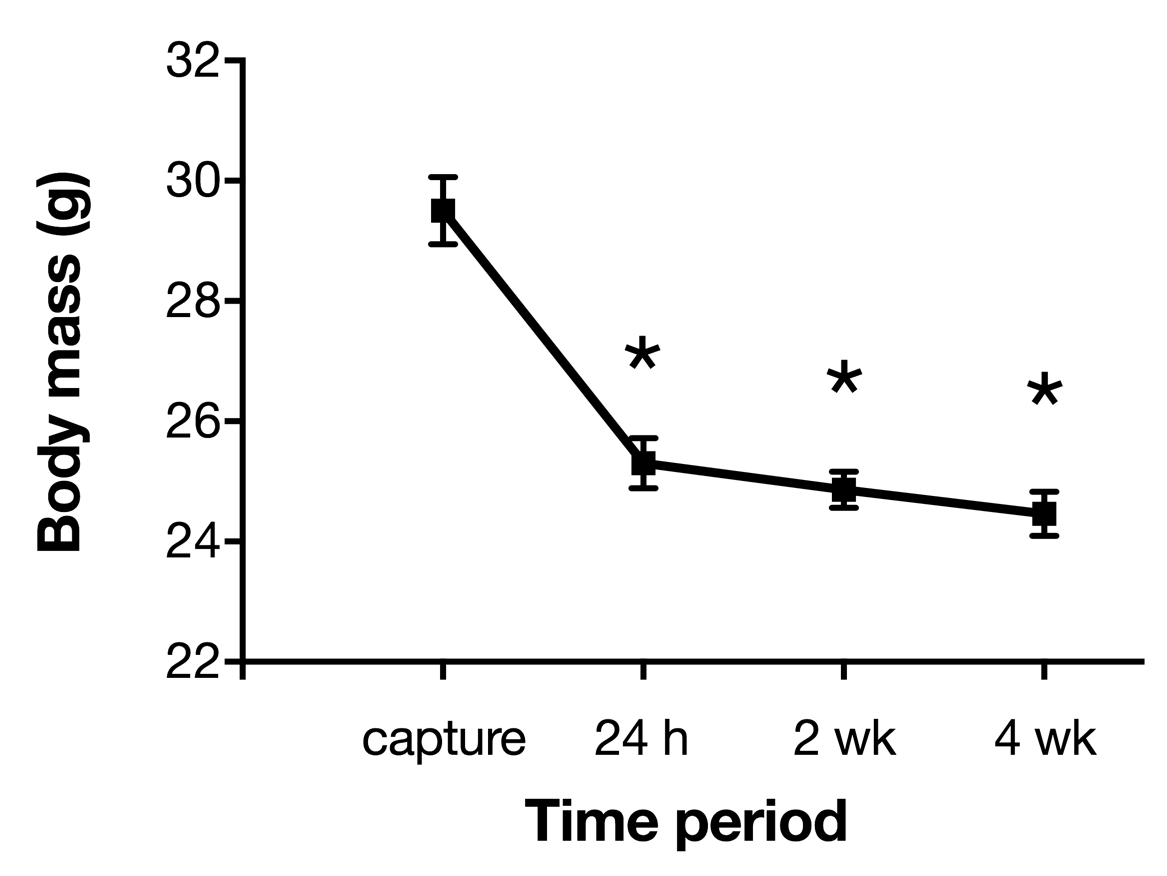


**Fig S2.** Wild house sparrows (*Passer domesticus*, n=15) lost significant body mass after 24 h in captivity, and there was no further significant change in mass over the next 4 weeks in a laboratory setting. Values are presented as mean ± SEM. Stars indicates time periods with significantly decreased body mass compared to capture (p<0.05).


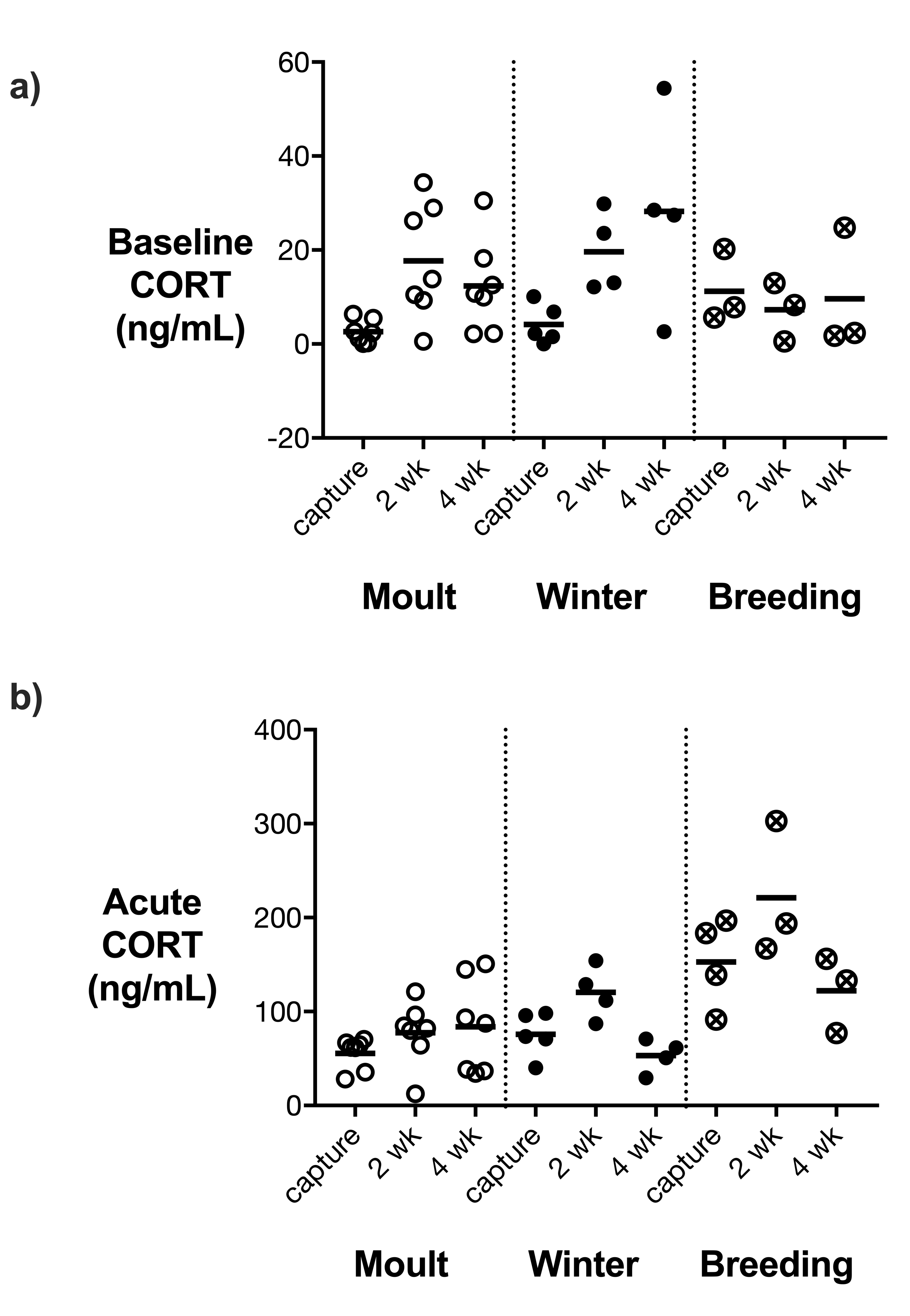


**Fig S3.** Plasma corticosterone concentrations in house sparrows (*Passer domesticus*, moult: n=6; winter: n=5; breeding: n=4) at capture and after 2 weeks and 4 weeks in a laboratory setting, displayed as individual data points to show individual variation. Baseline samples (a) were collected <3 min from time of capture or from entering the bird room; acute samples (b) were collected after 30 min of standardized restraint stress. Lines indicate mean values; see Fig 4 for more details.


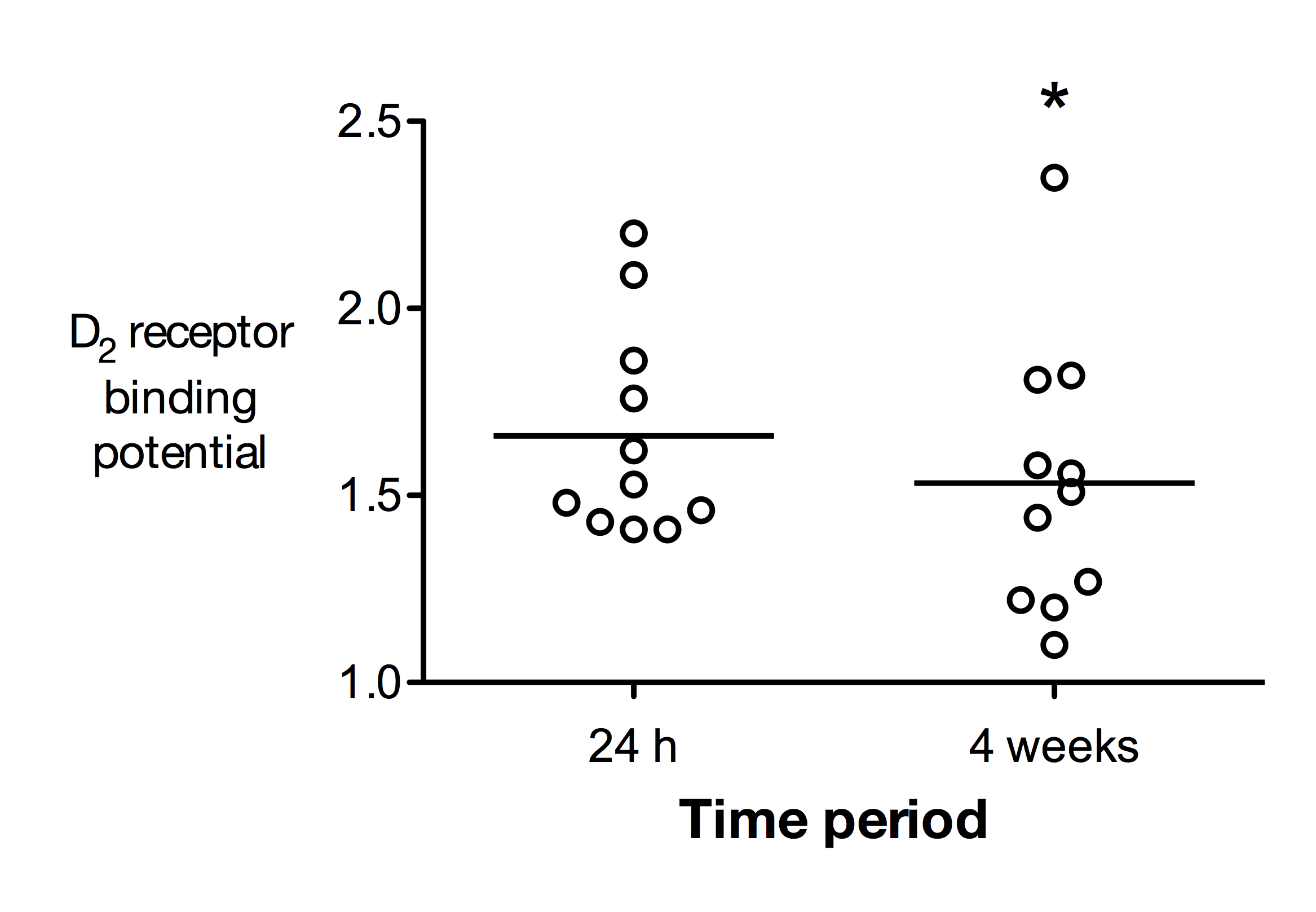


**Fig S4.** D_2_ receptor binding potential (BP) in striatum of house sparrows (*Passer domesticus*, n=11) 24 h after capture and after 4 weeks in a laboratory setting, displayed as individual data points to show individual variation. Lines indicate mean values; the star indicates significantly decreased D_2_ receptor BP compared to 24 h post-capture (p<0.05).

**Fig S5**. Behaviour of wild house sparrows (*Passer domesticus*, n=15) after 48 h, 2 weeks and 4 weeks in captivity. Values are averaged across multiple observers from two 1 h videos that were recorded in the morning and evening of each time period, and are presented as mean ± SEM. Stars indicate time periods with significantly different behaviour compared to 48 h post-capture (p<0.05); see Table S1 for results of the statistical analysis.


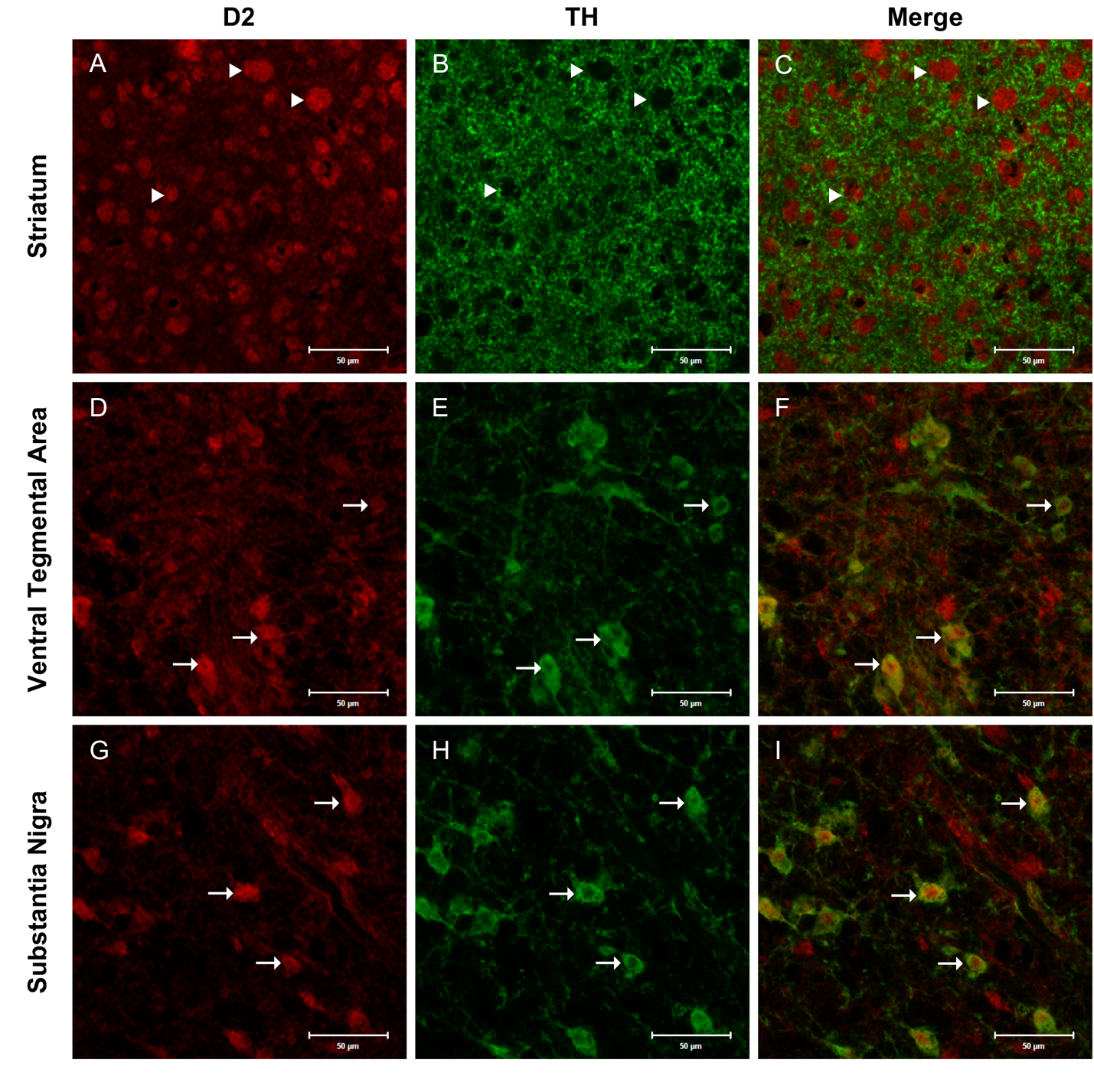


**Fig S6**. Representative double-immunolabeled brain sections from house sparrow striatum (top row; images A, B, and C), ventral tegmental area (middle row; images D, E, and F), and substantia nigra (bottom row; images G, H, and I). D_2_ receptor-labelled cells are seen as red (left column; images A, D, and G), tyrosine hydroxylase (TH)-labelled cells are seen as green (middle column; images B, E, and H), and co-labelled cells are seen as yellow in the merged image (right column; images C, F, and I). Arrows indicate examples of co-labelled cells and arrowheads indicate cells singly-labelled for D_2_ receptors. Scale bars indicate 50 µM.

**
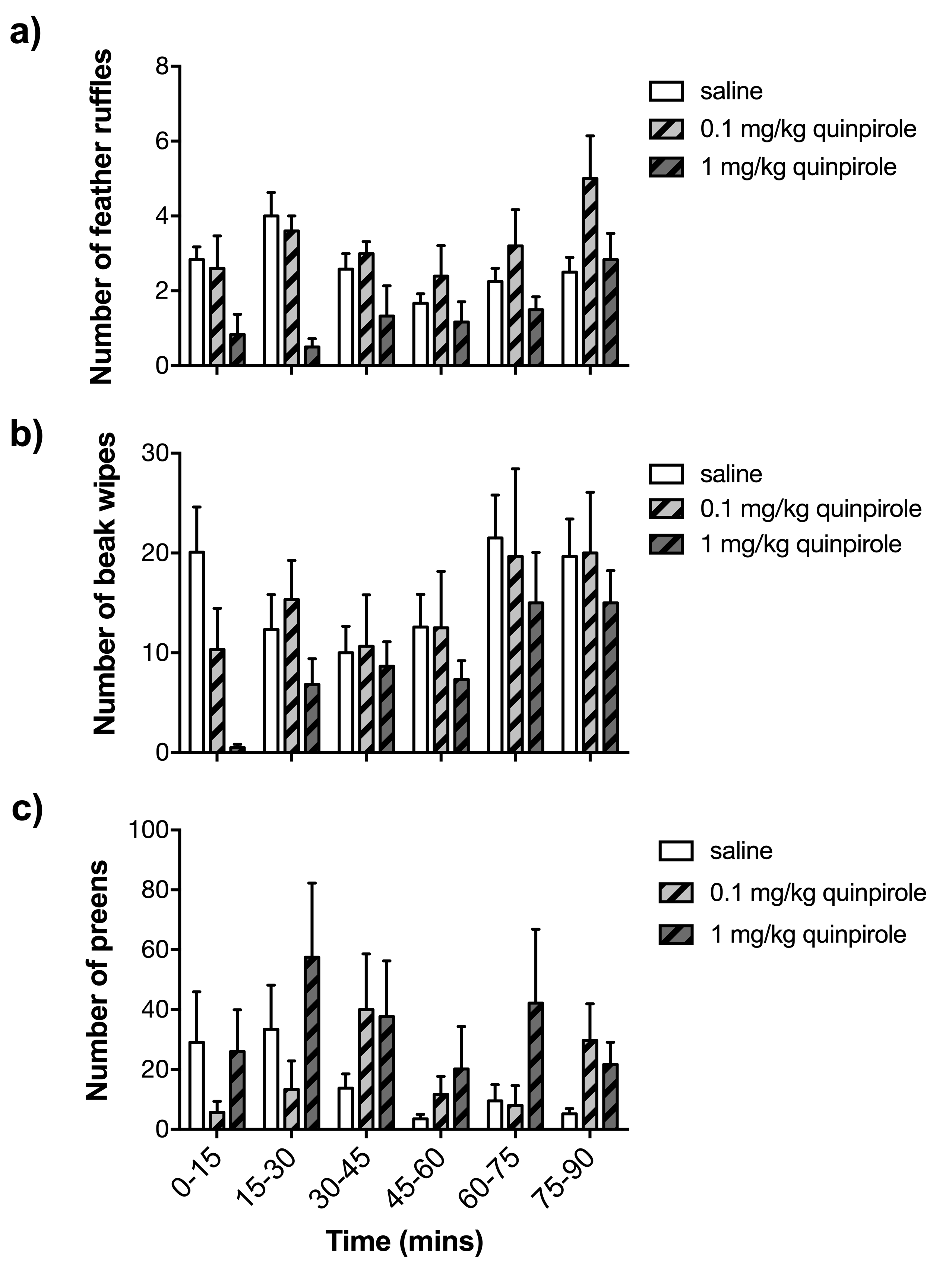
**

**Fig S7.** Administration of a 1 mg/kg dose of a D_2_/D_3_ receptor agonist (quinpirole) to house sparrows (*Passer domesticus*, n=6) significantly decreased the amount of feather ruffling (a) that captive birds performed compared to their response to a vehicle control (saline) and a 0.1 mg/kg dose. There were also significant effects of time period post-injection, and a time period*treatment interaction. 1 mg/kg quinpirole also significantly decreased beak wiping behaviour (b) compared to a saline control, and again there were significant time and time*treatment interactions. Preening behaviour (c) increased in response to 1 mg/kg quinpirole compared to a saline control, but there was no significant effect of time or an interaction between time and treatment. Values are presented as mean ± SEM, and shown as raw data even though statistics were run on transformed data. See SI Methods and main text (Results) for more details.

**Fig S8**. Western blots of the antibodies used for D_2_ receptors (D2; Panel A) and tyrosine hydroxylase (TH; Panel B). Protein samples were run undiluted. A single band for D_2_-like receptors is present at <100 kDa. A single band for TH is present at 60 kDA. Arrows indicate bands. Raw images were cropped to only show the ladder and protein lanes. No lanes were cut or spliced together. Contrast of each image was adjusted for optimal band visibility (simply to show the size and number of bands, not for quantification purposes) using ImageStudio (v. 3.1.4, LI-COR, Windows 7). See Supplementary Methods for interpretation.

**References**

1 Lessells, C. M. & Boag, P. T. Unrepeatable Repeatabilities: A Common Mistake. *The Auk* **104**, 116-121, doi:10.2307/4087240 (1987).

2 Balthazart, J., Castagna, C. & Ball, G. F. Differential Effects of D1 and D2 Dopamine-Receptor Agonists and Antagonists on Appetitive and Consummatory Aspects of Male Sexual Behavior in Japanese Quail. *Physiology & behavior* **62**, 571-580, doi:<http://dx.doi.org/10.1016/S0031-9384(97)00163-7> (1997).

3 Reiner, A., Perkel, D. J., Mello, C. V. & Jarvis, E. D. Songbirds and the Revised Avian Brain Nomenclature. *Ann N Y Acad Sci* **1016**, 77-108 (2004).

4 Sesack, S. R., Aoki, C. & PIckel, V. M. Ultrastrucural localization of D2 receptor-like immunoreactivity in midbrain dopamine neurons and their striatal targets. *J Neurosci* **14**, 88-106 (1994).

5 Kubikova, L., Wada, K. & Jarvis, E. D. Dopamine receptors in a songbird brain. *J Comp Neurol* **518**, 741-769, doi:10.1002/cne.22255 (2010).

6 Nixdorf-Bergweiler, B. E. & Bischof, H.-J. *A stereotaxic atlas of the brain of the zebra finch, Taeniopygia guttata, with special emphasis on telencephalic visual and song system nuclei in transverse and sagittal sections. Bethesda (MD)*. (2007).

7 Amlaiky, N. & G Caron, M. *Photoaffinity labeling of the D2-dopamine receptor using a novel high affinity radioiodinated probe*. Vol. 260 (1985).
